# What a Dentist Saw in Examining Five Hundred Crania

**Published:** 1895-09

**Authors:** M. H. Fletcher

**Affiliations:** Cincinnati, Ohio


### THE DENTAL REGISTER.

VOL. XLIX.] SEPTEMBER, 1895. [No. 9.

### COMMUNICATIONS.

### What a Dentist saw in Examining five hundred Crania.

BY M. H. FLETCHER, M.D., D.D.S., CINCINNATI, OHIO.

Read before the Mississippi Dental Society, April 18, 1895.

*Mr. President and Gentlemen:*

In presenting this subject according to the program, it is not my intention to go into detail of any of the pathological conditions of the tissues of the mouth, but to offer to you a few facts which have impressed me during the little work I have done in examining crania. This work was begun with special reference to the study of the antrum of Highmore, and for the purpose of determining if possible what relation the teeth might have to the diseases of that cavity, since it is claimed by all the authors familiar to me (excepting Dr. E. S. Talbot), that antral trouble comes more largely from the teeth than from an other source. In the examination of five hundred crania, making one thousand antra, my conclusions were that the teeth were as often, and probably much oftener, made pulpless or otherwise pathologically affected by diseases of the antrum, than the reverse; the reasons for which were set forth in a paper read before the American Medical Association two years ago at Milwaukee, to which I refer you. In pursuing these examinations I found it difficult to confine myself strictly to the points in question, because of so great a number of most interesting conditions which presented themselves for a dentist's observation. In fact the title of this paper could as properly be, *what a dentist did not see*, in the examination of five hundred crania, as what he did see. It was most exasperating to find such numbers of interesting conditions, both pathological and normal, realizing that the time at command was not sufficient to accurately pursue even one subject—an experience corroborated by Dr. E. G. Betty.

In pursuing such work, I was particularly struck, as others have been, with the fact that almost every lesion which a dentist is called upon to treat, leaves its mark in some way on the hard tissues of the jaws or the teeth; I will mention most of them, and ask if I am not right. Of course all the normal conditions of the hard tissues and their histology can be studied.

In pathological conditions you may study:

Faulty development or osseous deformities of the head, jaws and teeth; certain features of the development of the hard tissues; tumors of bone, necrosis and caries; faulty development of the teeth in enamel; of Dentine or Cementum, malformed teeth either permanent or temporary; irregularities of teeth as to position; united teeth; fused teeth; supernumerary teeth; supplemental teeth; nodular teeth; tumors of teeth; syphilitic teeth; calcic deposits of all varieties; the effects of calcic deposits upon the bone, and its effect upon the teeth.

In the teeth themselves you may study:

Caries; necrosis; abrasion; erosion; fractures; hyper-cementosis; exposures of the pulp, indicating pulpitis in all its forms; all forms of new growths of the pulp chamber.

In the diseases of the alveolar process:

Alveolar abscess; necrosis and exfoliation; absorption or phagadenic affections; hypertrophy.

Diseases of the antrum, and the relation they may or may not have had to the teeth.

These and many others points of practical interest to the stomatologist may be studied with great advantage by the examination of crania.

You may say there is nothing new about this, that we all knew it before, and that it has been gone over by the Examination Committee of the American Dental Association, by Dr. E. G. Betty and others, and the results have been published, most of which is true I admit, but who have been the greatest gainers by these examinations? Those who have done the work, without doubt, for *they* have come in direct contact with the objects and phenomena, and received the greatest benefit thereby.

There are important features to be observed, however, which I have not yet seen published. *One for instance*, is that the dental follicles holding the crowns of the superior molar teeth (in normally shaped antra) are usually formed at the expense of that cavity, each follicle being covered with a dome of bone which protrudes into the floor of the antrum, while the tooth is being formed and pushed into the mouth; this dome flattens out and entirely disappears by the time the tooth is completed, excepting in rare cases; this seems a perfectly natural result, when we remember that the alveolar process is only formed after the eruption of the teeth, its position and shape being entirely governed by the position and shape of the teeth; there is, then, no other place where the crowns can find space enough for their development but the one mentioned at the expense of the antrum. The finding of some of these domes in the floor of the antrum, in connection with some bony processes, may account for the description by anatomists, to the effect that the roots of the molar tooth protrude into the floor of this cavity, and are either bare or covered with bone, forming numerous bony tubercles corresponding to the apices of the sockets of the teeth; other authors taking these as authority have perpetuated this statement until it seems the prevailing opinion that this is the normal condition.

Zuckerkandle pictures these in a normal skull, and most works on anatomy, which I have seen, speak of it as being the normal condition; Gray, Leidy, Quain, Holden and others do so, in fact I have seen no exception to this description in any work on anatomy; but over one thousand antra according to my examinations *show it only five times*, and one of these specimens I pass about for your inspection.

As to another point, I quote the following from Dr. Boderker's late work on "Anatomy and Pathology of the Teeth;" he says, "the apices of the roots of the second biscupid and the buccal roots of the molars are in contact with the floor of the antrum." This is given without exception or qualification; an examination of a few sections through the alveolar process and into the antrum at this place, will, I think, show this to be the exception rather than the rule; since these specimens are more common, according to my own observation, and I believe it to be normal; the apices of the roots of the molars do sometimes come in contact and often perforate the floor of the natrum as shown in specimens, but I find this condition the exception rather than the rule.

As to the second bicuspid, I have very seldom seen it in contact, or even pointing directly toward the floor of the antrum, and a drill taking the direction of the axis of the tooth would most likely run a quarter or half inch before reaching even the anterior wall of the cavity, and just as likely miss it all together, or in some cases would probably reach the floor of the nose.

These may seem like minor or unimportant points, but since we must have the anatomy and osteology of these parts described, for the benefit of those who are likely to deal with pathological conditions in this region, the description had better be right than wrong, and dentists or rather stomatologists had just as well have the credit of doing it right as any other set of men, hence, the necessity of investigating and discussing the subject. I have never seen a description of these features in any text-book based on a series of examinations, but have seen such statements as referred to, over and over again, and made without exception or qualification, the following, as an example is copied from Gray's last Edition, describing the antrum or maxillary sinus, he says, "projecting into the floor are several conical processes, corresponding to the roots of the first and second molar teeth; in some cases the floor is perforated by the teeth in this situation;" and *he* quotes Mr. Salter on "Abscess of the Antrum" as authority. If the points referred to above are of a sufficient moment, and need correction, let us present sufficient proof, and have the correction made, if the statements herein made are based on error, then *I* shall be exceedingly glad to be corrected.

Another fact of great interest to me was, that showing an almost total absence of dental lesions of any character in the mouths of Northern Indians and Esquimaux. In forty-eight Alaska Indians, only two abscessed teeth were found, and these teeth seem to have been broken rather than decayed. In thirteen Esquimaux, no abscesses and no decay was found in the whole *sixty-one skulls*; there were exceedingly few with calcarious deposits upon the teeth; and no cases where the teeth had apparently been lost from this deposit and its consequent results. The cases of abscesses in these tribes show about 3 per cent, whereas, the cases of abscessed teeth in the Mississippi Valley Indians proved to be about 30 per cent; these were all upper molars, but the comparative per cents would probably remain about the same if all abscesses had been counted.

The loss of teeth from tartar and its consequences in the Mississippi Valley Indians was simply enormous; having taken no statistics on this point, I cannot give numbers and per cents, but speaking from memory, I should say that the loss from tartar in the Mississippi Valley tribes must have been found in 33 per cent of cases. Great quantities of tartar still clinging to many of the teeth, and numbers of cases showed unquestionable evidence of the teeth having been partially, and in many instances totally lost from phagadenic diseases; which, doubtless, were brought about by excessive accumulations about the teeth.

These facts are significant for the reason that the food of the two classes or nations were so different; the northern tribes living almost entirely upon meats and oils, which are free from grit, and need but little mastication; while the tribes living in temperate or torrid zones lived more largely upon the vegetable diet, containing more or less grit, then the vegetable substances usually need more mastication than meat before they can be swallowed. If I tried to account for the difference in quality of structure or loss of teeth in these people of such different habits, it would be after the above manner, and that after long lapses of time heredity would cut quite a feature, for no doubt generation after generation of these tribes lived under the same environments.

The chemical difference between animal and vegetable foods may cut some figure, but I believe the mechanical feature is primarily the greater factor.

As to diseases of the antrum, it seems to me that these people were remarkably exempt, when we consider that they could have had no surgical or medical attention; and also that about 25 per cent of them had abscessed upper molars. This fact is significant, too, when it is claimed by the majority, and, in fact, nearly all authors, that diseases of the antrum come more largely from this class of teeth than any other source. This series of examinations show that out of the 252 cases of abscessed upper molars, only 12 perforated the antrum. This would seem a remarkably small number and count as a point in favor of claiming that abscessed teeth do not cause antral trouble as often as most authors maintain they do, in comparison with other causes of inflammation of this cavity.

My reasons for believing that the teeth are, and may often be, affected by diseases of the antrum, are strengthened by considering a certain feature of the anatomy of the parts, mentioned by Gray, and omitted by most other anatomists—namely—that, "in some cases the floor is perforated by the teeth in this situation." I found this to be the fact in twenty cases showing that about 4 per cent of persons have, normally, nothing covering the apices of these teeth but mucous membrane; I here would say that the statistics on this particular point *are not accurate*, on account of the inability to see into the antrum, in an unbroken skull. Many skulls were broken, however, so that examinations could be thoroughly made on this point, as well as others; and when they were not broken, the sense of touch was used to determine the presence of bony processes, septa and the general form of the cavity, and the normal openings above the teeth as far as possible. It was also observed that these normal openings occurred where the floor of the antrum was comparatively flat, and not where there was a conical process, and that those cases where the conical process occurred were almost invariably covered with a considerable thickness of bone.

These conditions being present, it would seem a natural result, when the mucous membrane of the floor of the antrum becomes broken down, for the blood and nerve supply of teeth so perforating the floor to be interfered with, and possibly entirely destroyed, since the apical foramen of the teeth must be exposed to these destructive influences. In cases of occlusion of the osteum—maxilare and other openings into the nose, the antrum may become tensely engorged, and under this condition, if there be no bony covering to the apex of a tooth, it would of course be more or less driven from its socket and become very sore to the touch, or occlusion with its antagonist, under such conditions I can imagine the teeth having been blamed for the trouble, since it, or they, would be exceedingly tender on percussion, and one under these circumstances would be most apt to say, "there is your exciting cause in that tooth."

It is my belief that if accurate statistics could be had, they would indicate that the exciting causes of diseases of the antrum would show, say, 10 to 1 in favor of intranasal disorders. I make this statement, taking into consideration the larger per cent of teeth that are known to perforate the bony floor of the antrum, for my observations also show that a much larger number of teeth are denuded of bone, on the buccal surface of the alveolus, hence, the *large preponderance* of abscessed teeth which discharge in this locality, a place where one familiar with the disease invariably looks for a fistulous opening. In this situation, if the bone is not entirely absent in some spot, it is so thin that it offers to internal pressure the least possible resistance of any other part of the sockets of the teeth.

Other points of much interest to us as specialists could be considered, but since I took no accurate figures on them, I will defer their discussion until close and more careful observations can be made.

### DISCUSSION.

Dr. Wright: Dr. Shields has an interesting case, the relation of which will occupy but a few minutes of the time of the society; he was once a dentist but from physical and mental causes (laughter) was incapacitated and has gone into another specialty of medicine; he is still interested in our profession, and as it will be to the interest of the society, with your permission I move that we liston to him for a few moments before Dr. Cravens starts the avalanche.

There being no objection, Dr. Shields took the floor.

Dr. Edward Shields, of Cincinnati: It has been some time since I interested myself actively in the dental profession, but up to the present time I have not lost all of the interest that I once cherished for the profession. A very interesting case of actinomycosis in the pulp on the teeth came under my observation a year ago last March in this manner: A patient was recommended to Prof. Albert's clinic for diagnosis of a swelling, occuring on the right side of the lower jaw. By mistake he came into the dermatological department, I was there in the laboratory at the time, and the first assistant seeing that the patient had a swollen jaw, said to me, "you were once a dentist; remove the tooth." I did so, thinking I was dealing with an ordinary abscessed tooth; and out of curiousity I fractured the tooth, and was surprised to find an ossified deposition in the pulp; the age of the patient being seventeen I thought it was rather strange that an ossific deposit was to be found in the pulp. A few moments later the physician who referred the case to Prof. Albert's clinic came in, and I showed him the tooth, and told him I had removed it. He said, "that was not a case for you; that is a case of actinomycosis, and should have gone to Prof. Albert." I quickly gathered the fragments of pulp and tooth and took my pulp and macerated it, and put it under the microscope, and was gratified to find that this ossific deposit was nothing more than a mass of actinomycosis. As the tooth was not decayed in the least it led me to believe that the tooth was not the source through which the infection had passed, but that it was secondarily affected through the blood-vessels. The second case in question is one that Dr. Callahan has some knowledge of. In 1888 a physician of this city came to me with a swollen face and dead tooth, apparently perfectly sound otherwise no fissures decayed. I opened up the pulp cavity, and removed the pus, and treated the tooth by the usual methods then employed; and in due course of time the tooth began to die, and the pulp canal was filled with oxyphosphate of zinc and cotton—that is, the cotton saturated with the oxyphosphate. About six years later he again presented himself at my office with the first or second molar badly affected in the same manner. The first tooth was in the upper jaw. The second case was in the lower jaw—first, or second bicuspid, I forget which.

It was very loose, and I pushed it to one side, as you would a temporary tooth, and then took my forceps and attempted to remove the upper tooth. To my surprise I found that he had nothing but a crown, with two pins from the two oxy-phosphate pulp fillings remaining in the crown of the tooth. Six months later, on the opposite side I removed a tooth having the same characteristics; and Dr. Callahan, I think, had the pleasure of removing a tooth from this gentleman's jaw either this or last year. Now, as to the etiology, I must say, that I don't know—have not the slightest idea as to the cause. There is no specific history, and the only theory that I can think of giving is, and it is hardly possible, that of a thromba clogging the vessels and cutting off the nutrition of the tooth and consequent absorption.

PRESIDENT: We will now proceed with the discussion.

DR. CRAVENS: I am somewhat in the position that Dr. Frank Hunter said he was yesterday; he was designated to open the discussion on the subject of pyorrhœ, and he said he did not know anything about it. I suppose that the committee in the same spirit selected me, because I have paid very little attention to the antrum. However, the antrum is there, and seems to have come to stay; and since I was selected to discuss the subject I have tried to find out something about it, with the assistance of the encyclopedia and some other aids. (Laughter.) A long time ago an inspired individual said, "Seek, and ye shall find." We usually can find abundant evidences to our own satisfaction at least, to support any preconceived opinions we may have in regard to religion, politics or pathology, or any other matter. I know it is so in my own case. I can prove anything to my own satisfaction upon which I have formed an opinion. I am afraid that Dr. Fletcher, when he started those laborious researches, probably set his stakes ahead for five hundred skulls; now, I gather that from the title of his paper; he set his stakes for five hundred skulls, and with a determination to do or die in proving what he believed to be the truth. And every man who is seeking for what he considers to be the truth, and who has a determination to get at the truth, has to be inspired with the idea that he is to do or die, that he is going to find it—that's all! So that, perhaps, the essayist, to his own satisfaction at least, has proved his point. I am not going to quarrel with him on that. I agree with the essayist that in hunting for the causes or the etiology of antral troubles, entirely too much has been charged against the teeth, and that it is the exception, the very rare exception, for the dentist to observe antral troubles. I can only recall three, and I have been practicing longer than Dr. Fletcher by a few years; and perhaps one reason why I have not discovered something about the antrum, as he has, is because he has made a specialty of that; in other words, he has been seeking that. "Seek and ye shall find!" He has been seeking for specific information, as various other members of the profession have done, by a certain line of study. I want to compliment the author of the essay for his perseverance. There is not much encouragement, not much to excite enthusiasm, in searching for truth in Golgotha; and there is where he has been searching for it! He states that from what he has seen in those one thousand antra, he believes that the ends of the roots of molars projecting into those cavities are always uncovered. Is that right? (to Dr. Fletcher.)

Dr. Fletcher: Not always. I gave a per cent.

Dr. Cravens: I believe that if the Doctor had examined those skulls in the living subjects (and that would be difficult), I think he would have found his proposition a mistake. I believe that nature never shoved the apex of a tooth root into the Highmorian cavity, without a bony protection, however thin. She never completed the apex of a root of a tooth in the mucous membrane alone. The denudation of the apices, as seen by Doctor Fletcher, we cannot deny. No doubt he found that they were denuded, and by the very process given in my essay of yesterday in pyorrhœ (Riggs' disease), in my quotations from Dr. Dawbarn, where he attributes destruction of bone to a perfectly natural process, due to periostitis, it is what he calls "chronic non-infectious." It is utterly impossible for apical developement to proceed in the air. There is a rhinologist in Indianapolis (there are a number of them there), a Doctor Kline, who has made a special study of this subject. I had the pleasure of listening to an essay or report on that subject he made before a little assembly of surgeons. He said that in treatment the opening in the antrum must be made very free—I think he used a drill for this purpose, and he said it was $\frac{3}{8}$ of an inch in diameter. He did not have perhaps just the proper instrument, but he wanted something to make a hole to get a drainage of the antrum. I will cite here one case that came under my own notice about a year ago. It was not very profitable to me (may have been to humanity, however), the patient was an evangelist, a sort of a missionary for the Young Men's Christian Association, and he was totally incapacitated for his work by an antral trouble; a dentist in Indianapolis (not myself), had extracted a molar for him, which was very badly broken down; and, it seems, in his desire to get a sufficient hold, he had pressed upward, and had forced a root up into the antrum. I enlarged the opening until I could put the end of my little finger in it. The reason I did so was, that just before that I heard Dr. Kline state how large he made his openings. I gave the patient a syringe and told him what solution to use. He attended to it, and a short time ago a gentleman who is related to him told me the opening had healed up entirely and the patient was comfortable, and I suppose, grateful. It happened that on Tuesday morning I received by mail a copy of the *Lancet*, published in New York, of the February issue. There were a number of short articles of a surgical nature, and I glanced through them. One attracted my attention. It was an article by Dr. Scanes Spicer, on "Chronic Empyemia of the Antrum Maxillare." It threw a light on this subject of perforating the antrum. It says "after a crucial incision over the canine fossa and reflection of flaps, a large opening is made with a chisel and mallet." A dentist is a better mechanic than the ordinary surgeon; a dentist would bore the hole every time; but this gentleman gets there just the same. "A large opening is made with a chisel and mallet" (he places the patient under the influence of an anæsthetic) "in the anterior wall of the antrum, according to the methods of Dr. Robertson, as published in the *Lancet*, 1892." Great care is taken that the bone is chipped away down to the level of the floor of the antrum, and a groove established down the alveolus. The opening is large enough to admit the finger to explore the interior. The interior surface is now curetted thoroughly so as to remove every trace of soft fungous granulation tissue, abscess sac, polypi, cysts, necrosed bone, or other objectionable material. Instead of now closing the operation, I introduce the finger into the antrum to act as a guard, and pass Kraus' trocar and canule down the inferior meatus of the corresponding nostril well behind the nasal duct opening, and make one or two large perforations through the inner wall. Chips of bone are usually pushed into the antrum, and can be detached by manipulation. We have left now as well as the large opening in the anterior wall a permanent large accessory osteum maxillare in the inferior meatus of the nose. He goes on to say that he "packs anterior opening with creolin gauze, tightly, for twenty-four hours, to assure permanency of opening for drainage and douching purposes. Washes with solution of boracic acid three times daily with a syringe; also frequently has patient blow air through antrum from the mouth and through opening into the nose, this latter process induces movement of currents of mucous and other stale fluids in the antrum. Cure certain and healing regular, and reasonably quick." Now, in regard to opening the antrum, I am not trying to deal with all the Doctor's paper. I don't feel myself competent. In such an operation then, as draining, the prime object should be to secure better drainage; and the question as to the shortest route or the most direct route, perhaps, is entirely secondary. The main question is to secure better drainage, so I suppose that is the object of Dr. Spicer in making so large an opening. By the way, Doctor Fletcher, when you want to continue your corrections of anatomical descriptions given in the text-book, I hope you will succeed in getting them to say nasal fossa instead of canine fossa. Dr. Fletcher is to be commended on his able declaration against the prevalent custom by which our text-books are made, viz: quoting the statements of others and so perpetuating what errors have crept into the original writer's text, from one generation to another.

Dr. Betty: I did not know anything about the Doctor's paper until he sent it over to my office day before yesterday. I heard it in its entirety to-day for the first time. It was not my intention to criticise his paper, so much as to congratulate, not only Dr. Fletcher, but all the members of this society for having on their list of membership any man who has concluded to devote some time to original research.

Among the points that Dr. Fletcher has taken up is one that is new to me, in this: that the anatomical formation of that portion of the antrum in contact with the apices of the teeth being a source of or a guard against devitalization of the pulp, or acute chronic catarrhs of the antrum. I have forgotten whether the Doctor states in his paper or not, that it is very difficult in the great majority of specimens to see into the antrum at all. In this specimen, which he passed about, the interior of the antrum cannot be seen, unless an incision has been made upon one or the other of the aspects of the antrum, in order to get vision into it. This specimen has been cut in order to afford such an opportunity. In the various collections in the museums of crania, I will say, they are very, very jealously guarded. Any one from the outside, a student or investigator, who essays to use this material in any manner at all, is closely watched, almost searched, before he comes into the building, and pretty sure to be scrutinized before he leaves it—in fact, some specimens he is not allowed to look at, at all, for fear in the handling, some portion of the specimen that may illustrate some particular point, may be carried away, or that those examining it may be more or less careless and some accident may happen to it, the specimen may drop or something fall upon it, by means of which its value may be destroyed, its usefulness for the particular purpose for which it is kept, impaired. I was very much interested in this paper, and I am glad to see that Dr. Fletcher has made this point, which was new to me. Hereafter, when doing anything of the kind myself, I shall endeavor to include in the investigation a thorough examination of the antrum as it is possible to make, for the reason that it will have a particular bearing upon our practice in any such cases of antral trouble as may come under our supervision. Dr. Fletcher makes the point, I believe, that in 4 per cent of cases he found, where the apices of the roots projected through the floor of the antrum, there was covering those 4 per cent, a mucous membrane. I would like to add to that, that there must be, in addition to the mucous membrane, a sub structure of cellular tissue containing the nerves and blood supply to those apices; that must necessarily be there. I have therefore derived the inference that, supposing one of those teeth had penetrated the antrum, we will say from first to third molars inclusive, possibly in rare cases the root of the second bicuspid—although he has shown these specimens to the contrary—that a sudden blow or injury to one of those teeth would be a direct cause of probably some form of antral catarrh. In the first place, there would be an acute inflammation, resulting in catarrh, possibly self-discharging, of the products of inflammation—pus—into the nose, or sometimes out directly into the mouth, either through the opening made by the extraction of that affected tooth, or alongside of the periosteum, the space occupied by the pericementum, that membrane lining the alveolar socket, and covering the root of the tooth. Pus would naturally gravitate in that direction rather than accumulate on the floor of the antrum. There is an opening into the nose; usually, I think, it is opposite the floor of the first turbinated bone.

Dr. Wright: Are you going on the supposition that the patient always maintains the vertical position?

Dr. Betty: Not always. He does three-fourths of the time. That is a very important point brought out in Dr. Fletcher's paper, one that I shall adopt hereafter in any further examination I may make. There are two or three other points that I want to refer to. Dr. Cravens was inspired upon this occasion to make a statement to the effect that he does not believe nature ever intended to shove the apex of a root in the Highmorian cavity.

Dr. Cravens: I said uncovered by bone.

Dr. Betty: I would like to ask Dr. Fletcher another point that is the reverse of this. Would a catarrh of the antrum, in such cases, say this 4 per cent of cases where there is a formation of the floor of the antrum, and nothing over-laying the apices of of the roots but the soft parts, would a catarrh of the antrum produced by continuity of tissue from nasal catarrh—could it result in the devitalization of any such tooth perforating the floor of the antrum?

Dr. Fletcher: I had one case in which every tooth—molar tooth and second bicuspid—were completely devitalized by engorgement of the antrum with pus. The bone was broken away in this case.

Dr. Betty: But it proceeded so far as to slough off the soft parts?

Dr. Fletcher: Yes.

Dr. Betty: And thence the devitalization of the tooth pulp as a consequence?

Dr. Fletcher: Yes.

Dr. Betty: That is the point I wanted to get at. I want to say, Dr. Fletcher, in all the papers on examination of crania, or examination of the jaws in the dessicated state, that is, the dry state, such as you see—these specimens on the table before you—and even in the tables I have gone over in journals on craniology and anthropology, I have seen no tables relating to the measurements of the antrum, or any of the sinuses, nor have I seen this especial point that you make relating to those protuberances or elevations on the floor of the antrum to accommodate the apices of the encroaching teeth; so that Dr. Fletcher, so far as I know, is the first one to have made a series of examinations of that kind. Hereafter we will see that it is also included, and furthermore, I would like to say that Dr. Fletcher's work brings him very closely to us, a statement that he makes in the paper somewhere, he asks the question, "who derives the benefit from these examinations?" "Who derives the greatest benefit from these investigations? Undoubtedly the one doing the work." Those who have done no work of this kind at all can have no adequate conception of the amount of time and trouble and worry there is, in order to gather together any data or statistics, no matter how small, in order to either confirm or disprove any particular point that may be mooted or called in question; for instance, as this one is here brought out in this discussion. Dr. Fletcher, as I stated, makes a statement that 4 per cent of cases of perforation of the antrum by the apices of the roots are accompanied by certain peculiarities; neither Dr. Cravens nor myself having made any similar examination are not prepared to either accept or controvert this statement. This indicates the necessity for each of us to undertake particular lines of original research in order that we may bring something of value to these and similar discussions. I say this, not especially with reference to this particular examination, or to show that the facial index of a race of people proves a certain intellectual capacity, but that such investigations afford some tangible results always, something that the reason can take hold of, data for analysis and comparison.

Furthermore, any such statements as made by Dr. Fletcher, when founded upon a series of investigations, must necessarily carry a weight of conviction owing to their positive nature as evidence collected in that manner, and brought before us so graphically that you cannot help being interested in further pursuing the subject. We are all benefitted by such studies, and the more so when they do not seem in line with previous experiences. If they substantiate former theories, well and good; but we are benefitted in any event. The one that first promulgates the theory will be benefitted; he makes the examinations, and his mind is thereby broadened and enriched; then he gives to us his 4 per cent conclusion; we are then 4 per cent in advance of previous thought in that direction.

Another point that Dr. Fletcher gave me this morning; or, possibly, I pointed it out to him; I have forgotten which; and that is, in making these investigations he was absolutely astonished, and so I admit was I myself, to find out how really little I actually knew of the anatomy and structure of the jaws, a portion of the human anatomy around and with which we are busy every day! We wondered that we knew so little as to the structure of the teeth, and how they are set in the jaw. You may use diagrams of crania and photographs and other means of graphically describing those parts; but they convey no idea at all comparable with the study of the actual specimen itself. You take up a skull and examine the mouth. Here in this specimen, all you see is this portion of the dental arch, three or four straggling teeth, and ten, fifteen or twenty holes in this arch; that is, apparently, all there is in view; yet when you come to analyze it, and consider its anatomy, and having learned its anatomy try to get some idea of its physiology, and after accomplishing that, in some measure try to get a further idea as to its pathology, you will find that the subject grows upon you to such a degree that it is almost appalling. And in a vast museum there is not only that one specimen, but miles, apparently, of shelves, rows upon rows, and you want to give up almost before you begin.

There is one other point in the paper that I would like to emphasize rather more than I have done. I want to ask this question for the benefit of myself, and, of course, through me for the benefit of others. "There are important features to be observed, however, which I have not yet seen published; one, for instance, is that the dental follicles holding the crowns of the superior molar teeth, in normal shaped antra, are usually formed at the expense of that cavity; each follicle being covered with a dome of bone, which protrudes into the floor of the antrum, while the teeth are being formed and pushed into the mouth. This dome flattens out and entirely disappears by the time the teeth are completed, excepting in rare cases. It seems a perfectly natural result when we remember that the alveolar process is one formed for the eruption of teeth, and its position being entirely governed by the position and aspect of the teeth. There is, then, no other space where the crowns can find space enough for their development but the one mentioned, at the expense of the antrum." I would like now to add to this, that we will have very likely to take the skull in *utero*, before there is any crown to the tooth, and before there has been any deposit at all in the *cul de sac*, or dental follicle in which the teeth begin, or germinate, before there has been any ossific deposit at all. That time must be along very early in fœtal life, because as early as the tenth week of intra-uterine life, the germ of the first molar of the permanent set of teeth makes its appearance. We will go along then until the seventh or eighth month; we will then have the bony structure of the cranium; I think there are sections made of that; it will be found in Flint's text-book on physiology, published some eight or ten years ago; there must, then, be a contour of the face, more or less definite, and underlying that of course under the cheeks, under the malar portion, is the projection of the malar process, of the anterior aspect of the malar process, and the superior maxillary that forms the outer and interior wall of the antrum. Now, then, Dr. Fletcher maintains that the teeth are formed at the expense of the conformation of the antrum, because the teeth are formed before three years of age, and in what we know and denominate as the alveolar process; and I can say, according to your definition, Dr. Fletcher, if I understand correctly, that the crown will appear somewhere between the floor of the antrum, between that and an imaginary line showing what will be the alveolar process of the future, when it is formed, as it will be, in the adult skull. Now, the particular bearing that I wanted to ask Dr. Fletcher, of this question, is why Dr. Mummery (?), in his examinations—and Dr. Talbot more recently—lays great stress, very great stress, upon the lateral measurements of the jaw being made opposite the first molar of the permanent set of teeth? Can you answer that?

Dr. Fletcher: I cannot.

Dr. Betty: I don't think Dr. Talbot can answer it, either. He gave me no adequate reply when I put the question to him in this society. If, as Dr. Fletcher states, a portion of the teeth are formed at the expense of a portion of the territory occupied by this cavity, the alveolar process must be formed subsequently to that, and—in other words, as a growth from the inferior process from both antra and on both sides; is it not so?—equally in harmony or in unison with the projection of the alveolar arch from the palatine process of the superior maxillary, and that is before fusion takes place between the two; because, if I am correctly imformed, the union between the two superior maxillaries does not take place until, I think, after the condyles of the femur have become ossified, and a bona fide part of the thigh bone; I think that occurs about the thirtieth or thirty-fifth year, does it not?

Dr. Fletcher: I do not know.

Dr. Betty: The formation of the face, contour of the features, as regards the upper two-thirds of the face, from the eyebrow down to the lower portion—this portion of the face (illustrating by gesture)—the anterior and outer aspect of the bony wall of the antrum enters as an artistic element in the configuration of the features, and also is a racial distinction between the different varieties or races and families of mankind. Now, Dr. Talbot, in his measurements has followed Dr. Mummery in making the lateral measurements of both jaws, upper and lower, from a point being the middle of the outer surface of the first molar teeth, so as to get the widest diameter. His only reasons for making this measurement is, that the first molar of the permanent set erupts entirely free and independent of any lesion whatever, of any dentition whatsoever, either first or second, independent of the first dentition, because it comes in and straightens clear posteriorly to the second molar of the deciduous set, and also precedes, necessarily, the second and third molars of the permanent set. Now, I have found that the second molars are just as free and independent in their eruption as the first one is; not so with the third molar, I would say that it is not so uniformly situated in the lower jaw, owing to the angle.

Now, then, Dr. Fletcher, if you will be kind enough to answer that question I will be obliged. (Applause.)

Dr. Fletcher: I am not sure that I understand the particular feature referred to.

Dr. Betty: That it is at the expense of the antrum, and has nothing to do with the subsequent formation of the alveolar process—that the formation taking place in the arch is at the expense of the territory usually occupied in that portion of the face, by both maxillary sinuses.

Dr. Fletcher: It seems the most natural result to me that this should be the place, as stated in the paper, for the formation of those crowns. As to the value of this place for measurements, it seems to me that variation can only be the rule, for various influences may cause the crowns to stand in or out of the arch; the anatomy of the parts is such that they can only form in the antrum and by the time the roots are formed, and the crowns be pushed into the mouth, the crowns may be pointed in almost any direction. According to our present nomenclature we only know the alveolar process as a bone which surrounds the teeth. In my examinations

I was struck with this, in examining the skull of a child, finding the second molar with a dome. Then I went back over some examinations I had made, remembering I had seen several skulls of young subjects; after that I conducted my observations having in view as well that particular feature. The formation of the teeth, as I say, determines the position of the alveolar process and determines to a great degree the width of the arch. If the bone is already formed, or is of such density and unyielding character that the teeth cannot take their normal places you have irregular teeth, as we well know. It is my opinion based on my observations, that we have no antrum in a child of six of any size, because the space is taken up by the dental follicles of the coming teeth. Taking out those follicles and clearing out the spaces that they occupied would in my opinion probably make the antrum perfectly conform to the contour of the face at that age; and the presence of the teeth in that locality as the crowns become developed and assume their normal size necessitates the widening of the face, and more space for their subsequent places, and in that way you have a broadening arch; the bones will all give to pressure; even down to old age, they will conform to a certain amount of continuous pressure; and this being the fact, as I understand it, gives an explanation of how the teeth do what I have suggested: that they not only broaden the arch, but they produce the bone, or the bone is produced to cover the places where they may find spaces to come through into the mouth.

Dr. Sage: I don't know whether there is any special significance in the statement of the paper, as quoted by Dr. Betty, that the alveolar process forms only after the eruption of the teeth, and I am not sure that my controverting the statement would be of any special significance in this connection. I am inclined, however, to call in question the statement that the alveolar process is formed only after the eruption of the teeth; according to my reading, in dentition the alveolar process forms coincident with the development of the teeth, and continuous with the eruption of the teeth, is absorbed and again forms and is again absorbed; and until its final form is absorbed no less than three times before the process of dentition is complete; that is my understanding of the matter. And finally, the alveolus of the permanent teeth forms, as Dr. Fletcher stated, regularly and coincidently with the eruption of the teeth, assuming the position to which the tooth in its eruption serves as a guide. I don't know whether there is any significance in Dr. Betty's statement or not. I offer this statement now merely to correct any misapprehensions.

Dr. Wright: Mr. President: if you found yourself appointed to discuss the question of "what a dentist saw in examining five hundred crania," without having any knowledge of what this dentist's peculiarities were, would you not think that much would be left to your imagination? Therefore I began to guess. At first I thought, is it possible that Dr. Fletcher is sentimentally philosophical? Has he taken those skulls in his hands and said, with Hamlet, "Alas! Poor Yorick! I knew him, Horatio"—(applause), or, is he more romantic and dramatic, and with Salvini, will he exclaim, "Oh, it is so good to be a skull—a numbskull!" (Laughter) These were two of my guesses. I did not know whether Dr. Fletcher was an anthropologist, or was interested in any special branch of the subject of anthropology, or was an ethnologist; I did not know whether he was a simple anatomist; and so I was surprised completely, when I got a little paper the other day—night before last—when I was awoy from my encyclopedia, and away from my books, and could not "post" on the subject—a subject which I did not know anything more about than does Doctor Cravens. (Laughter.) But there were two points in this paper that struck me, as they have Dr. Betty and Dr. Sage, and so I took notes vigorously on the paper, quoting: "There are important features to be observed, however, which I have not yet seen published. One, for instance, is that the dental follicles hold the crowns of the superior molar teeth, in normally shaped antra, are usually formed at the expense of that cavity, each follicle being covered with a dome of bone which protrudes into the floor of the antrum, while the tooth is being formed and pushed into the mouth; this dome flattens out and entirely disappears by the time the tooth is completed, excepting in rare cases." I want to ask whether this is a recognized fact; and if not, whether Dr. Fletcher can bring sufficient proof for his statement, as he claims it has not been published before? Dr. Fletcher speaks of only five in a thousand antra showing the tuberosities or bony tubercles corresponding to the apices of the sockets of teeth. Without my authorities I am not certain about it, but I think many of those present, Drs. Smith, Callahan and others, are under the impression, with myself, that dental anatomists have recognized that the tubercles corresponding to the teeth sockets are only exceptionally shown, and that it is not the rule. Another point: in relation to the position or relation of the roots of teeth and the floor of the antrum. Is it not clinically shown, in surgical operations (and that is the way we know most about it at present, as surgeons) that the antra can be frequently *easily entered* through the socket of an extracted first superior molar; and that even the second bicuspid socket does, in some cases, form an easy entrance into the antrum? Those are the two points that I have noticed.

I agree fully with the author that correct anatomical descriptions of these particular points, "based on a series of examinations," should come from the dental as well as from the general anatomist; and I wish to compliment Dr. Fletcher on the part he has taken in this work. At the same time I think as careful scientific men, we should demand more explicit proofs of the positions taken by the Doctor, before we dare to quote him as an authority. We want more exact data. We want to have diagrams and measurements. The facts are to be had—if not in the Smithsonian Institute, then somewhere else—for crania exist; but we must demand accurate statements of methods of examination, methods of measurement, methods of arriving at percentages, before we can be satisfied on either of these points.

Now, as the question has been raised by Dr. Fletcher. Craniology is a science that has occupied the attention of many earnest students of ethnology in many countries; and France, England and Germany have contributed an infinite number of measurements and studies in this line—the study of the races of men. My attention was first directed to this study of ethnology in 1876 by a work by Oscar Peschel, and at that time I began to appreciate the difficulties of the study, first in regard to the especial point of the differences in races—the skulls either long or broad, the facial angle acute or obtuse, the length of other bones, the proportions of upper and lower limbs, the hair, etc. etc., as distinctive features of race; the influence of habit and heredity on the infinite variations in these proportions. During the Civil War in the United States measurements in regard to the dimensions of the body extended to 1,104,841 men. How many tables of cranial measurements have we today to which to refer! Why, there are over one hundred and forty measurements now employed on each skull by anthropologists and ethnologists.

I wish Dr. Fletchor would pursue some first-class regular method of measurement of this one region of the cranial anatomy, as a dental anatomist, and give to the world *facts* that would make his name and his city (and his friends) famous among scientific fact hunters. There are two kinds of scientific workers: 1st. The fact hunter, who gropes away as patiently as anybody can, in his laboratory under ground, everywhere under the broad heavens, to gather facts! 2d. Those who draw conclusions from facts thus so patiently ascertained.

A fact is a glorious thing to present to the world, to leave as a legacy. Think of what it means in this age of speculation and hasty deduction from flimsy falsehoods, in medicine and in dentistry and in general science!

In regard to the other observations of the author in his examinations of skulls, they are, as he well says, of intense interest and advantage to him who makes the examinations. I have enjoyed that pleasure in various places, in some old churches on the Continent, about Switzerland, in Alpnack, in Olton, at the convent of Mariastein, and where the skulls from neighboring battlefields have been gathered and placed on shelves, row after row, to the number of hundreds and thousands, in the vaults under those old churches. I observed these, as Dr. Fletcher has his five hundred, and have read in the lesions of these osseous histories, the aches and pains of the original possessors of the ghastly death's heads. But these were not scientific observations on my part. They were made for my own pleasure as a dilettante, and not as a fact hunter; and I have never felt called upon to offer any remarks, written or spoken, on the results, except in private conversation. I felt that I should have had a definite object, and should have pursued that object eagerly and earnestly, as Goethe did in his search after the mid-jaw in man. How many hundreds of skulls he must have hastily glanced at in searching for this one little point? And he found it, and was happy, for a great problem in anthropology was solved by its discovery. Ruskin says, "The more I think of it, I find this conclusion more impressed upon me, that the greatest thing a human soul ever does in this world is to see something, and tell what it sees in a plain way."

Dr. Smith: Some ten years ago I found in our dissecting room an inferior maxilliary that presented this peculiarity; it seemed as though the third molar had penetrated the inferior dental canal. I took the specimen to Chicago, and had it with me at the American Dental Association, and showed it to Prof. Atkinson, and called attention to the fact that I thought it seemed as though the root of the third molar had penetrated the inferior dental canal in its development. He looked at it, and was very cautious about expressing an opinion; did not say very much. He then said, "you ought to be very sure when you make an assertion of that kind. I doubt very much the entire absence of bone." If the position taken by Dr. Fletcher is true it may account for certain lesions—phases of facial neuralgia. I doubt it very much. It has set me to thinking, this question coming up since I have heard the paper read; I doubt very much if the root of a tooth ever penetrates the antrum. You must have a thin covering of bone, as Dr. Atkinson intimated. The bone of the antrum is proliferated by its own periosteum, and does not depend on any tissue of the teeth at all. If you take those skulls and examine the roots you assume that the roots never had a covering of bone, but they may have had, even though it was as thin as paper. If you examine those skulls where it is denuded the bone is so thin that in the dry state it falls away, and that is all there is about it. An observation made on a dry skull is nothing. We ought to be very cautious, as Dr. Atkinson said. We should be very slow to assume. If it is true that those roots will penetrate the antrum it will account for a great many of those cases of facial neuralgia, and we ought to know it.

Dr. Sage: Since Dr. Smith has spoken it occurs to me to say one word more. I was not aware at the time, yesterday, that I was committing the grave offense of talking forty minutes. You should have called me down. I think it is Jno. Tomes who speaks of the close approximation of the roots of the first permanent molar to the inferior dental canal as frequently accounting for those obscure cases of neuralgia which arise. I think it is Richardson, also, who cites an instance of the perforation of the walls of the floor of the antrum, in which he found a root, a cuspid had penetrated the anterior wall of the antrum.

Dr. Smith: In the normal condition, if the root is exposed, it will account for these lesions; I assume that has not been proven, and is hardly physiological.

Dr. Fletcher: In regard to this point that has been largely discussed, I cannot go through all that has been said. It seems to me perfectly natural that we may have the apices of these teeth denuded of everything but soft tissue, from the fact that on the roots of those teeth we have the pericementum and the mucous membrane covering it. You have in that exactly what bone has—it is covered with periosteum. This periosteum, if I know the anatomy and physiology of other parts of the body, in that particular position may be continuous over the floor of the antrum into the socket of the tooth and over the apices of these teeth. My examinations led me to believe that in a normal condition those teeth were covered with nothing but soft tissues. They could not be denuded entirely of everything but the mucous membrane—I should have qualified that by saying soft tissues—which includes peridental membrane, and over that the mucous membrane on the floor of the antrum. So that it seems to me a perfectly natural condition to find there, normally.

Dr. Smith: Without previous disease of the antrum? I mean any character of lesion?

Dr. Fletcher: Yes. In the examination of the crania you may see that bone had not been broken away. If you will take the pains to examine them closely, those two I showed you in which the apices of those teeth perforated directly through the flat floor of the antrum you will find no more space than sufficient to accommodate the peridental membrane, that is the space that it usually occupies. In examining them closely you will find that it has not the appearance of broken bone. It seems to be a perfectly normal condition; consequently, I reason as I do about it.

DR. SMITH: I will ask you a question: If that is the normal condition why do we have those upheavals over the ends of the roots?

DR. FLETCHER: That is a question I don't think anybody can answer. Why are some men bow-legged? You may say it is rickets, or anything else.

DR. SMITH: I would hold, then, one is the normal condition and the other is not. Why should the one be covered and in the other be denuded entirely?

DR. FLETCHER: Why, I could not say. I used the word normal; I should say physiological; there can be no question, then, about it.

Through personal illness and illness in my family I could not get at this paper in time to give it to the gentlemen appointed to discuss it sooner; so I wish to apologize to them for the delay. However, I feel very grateful and highly complimented in what has been said. One or two points, I think, Dr. Cravens spoke of in regard to selected cases. I don't see the Doctor here now; but if he will go with me to the Army Medical Museum and attempt to select special skulls out of 3,000 cases in a limited time, I know he will agree with me that it would take longer to select than to take them as they came. In fact such selection would take longer than the examination. The examination I will say (since that has been hinted at, seriously or otherwise), was seriatim, as they came in the cases, each one numbered by a private number, and also by the museum number. I can give the No. of every skull I examined; and you can go and see them for yourselves. I wish some of you would do so, gentlemen.

In regard to the matter of establishing facts, I can say, nothing would please me better than to have confreres associated in this work. I expect to continue it as time and opportunity favor, and should be glad to be shown mistakes. I have a mania for looking after facts, and am one of those fellows that Dr. Wright spoke of. The paper has been written with the object of bringing the subject before the profession, and if possible to stimulate enough persons to go at this work to get the statistics necessary, spoken of by Dr. Wright. There is no reason why we should not do it, and a great many reasons why we should do it.

President: I suppose we may regard the discussion as closed for the present.

On motion, bills in the hands of Dr. Callahan, amounting to $46.85 were ordered paid by the Association.

Dr. Callahan: I hold in my hand a card of regret from Dr. John S. Marshall. I would also remind you that as yet no provision has been made for election of officers for the ensuing year.

President: The subject next to be presented is a demonstration of Electric Oven and Appliances for accurately determining the heat, by Dr. L. E. Custer, of Dayton, O.

Dr. Custer thereupon proceeded with the demonstration, but as the exhibition would be unintelligible here without proper illustrations and figures, the stenographer made no attempt to report the same. The exhibition of the oven was very interesting to all present.

The Committee on Membership, Drs. Emminger and Welch, reported favorably the names of Drs. J. A. Stein, of Ripley, O.; F. M. McCarthy, of Shelbyville, Ind.; and W. B. Chambers, of Newark, O., as candidates for membership. On motion, the rules were suspended, and the names reported were accepted as members, and announcement made by the Secretary.

The Committee reported the following as delegates appointed to attend the American Dental Association meeting, to be held on the first Tuesday of August next, at Asbury Park, N. J. viz: Drs. J. E. Cravens, A. P. Bollinger, H. C. Matlack, W. S. Locke, Frank Sage, W. B. Chambers, W. D. Phillips, Sarah S. Harris, Jessie Dillon and Viola Swift.

President announced that the next order of business was the election of officers for the ensuing year.

On motion of Dr. Mollyneaux, the rules were suspended and Dr. Wm. V. B. Ames, of Chicago, was unanimously elected to serve as President of the Mississippi Valley Association of Dental Surgeons for the years 1895-6. The Secretary was instructed to cast the ballot accordingly, which being done was formally announced by Dr. Taft, in the Chair.

The remaining offices were then separately filled by the same process and the results in each case formally announced by the Chair, as follows: First Vice-President, Dr. J. E. Cravens, Indianapolis; Second Vice-President, Dr. H. C. Matlack, Cincinnati; Treasurer, Dr. F. A. Hunter, Cincinnati; Corresponding Secretary, Dr. W. S. Locke, Cincinnati; Recording Secretary, Dr. H. T. Smith, Cincinnati.

The Chair: What other officers are elective, Dr. Callahan?

Dr. Callahan: I think that is all. The Executive Committee will be appointed by the President.

The Secretary: There is usually a committee appointed on publication of the proceedings of the session.

The Chair: Have you all the papers?

The Secretary: The stenographer and I have all of them.

Dr. Callahan: Then, I presume, the committee to be appointed on publication will take charge of all the papers and the stenographer's report of this meeting.

Dr. Taft: I presume so. I will appoint Drs. C. M. Wright and Frank A. Hunter as a committee to conduct the newly-elected presiding officer to the Chair.

The committee thereupon escorted the newly-elected President to the rostrum, where he was received by Ex-President Taft and introduced by Dr. Hunter as follows:

Dr. Hunter: It affords me pleasure to present to you the "heir apparent," although we see he has no hair apparent.

President Ames: You would naturally expect me to be "covered with confusion." I thank you very much for the honor you have conferred upon me; all I can say is, that the only regret that I feel at the present moment, and which I experience from day to day is, that life is too short for each and every one of us to look forward to meeting with this Association for the next fifty years of its history. I hope to be an attendant for very many years to come, and that the next session can be made somewhere nearly as great a success as the present one. Thanking you again for the honor conferred, I will give my best effort.

Dr. J. Taft: Mr. President and gentlemen: I feel that I cannot take a seat without expressing to you my very great gratitude indeed, for the success of this meeting. I hear from every one nothing but satisfaction for the manner in which this work has been done, yesterday and to-day; and for this you are very largely, indeed almost wholly, indebted to the Executive Committee, who have been untiring in their perseverance to secure the success of this meeting; and it is due to them that we have had such a pleasurable time during these two days.

Personally I must express to you my warmest and sincere thanks for the manner in which you have carried this work through, and for the way in which I have been sustained, in guiding your proceedings—although, so far as guidance was necessary it seemed to go of itself, without much guidance. But I wish to express to you my sincere thanks for your support and co-operation during the two days past.

On motion of Dr. C. M. Wright, a vote of thanks was extended by the Association to the retiring President, and officers of the Society for the able work during the session, and before the session, in affording the members the pleasure of this meeting when last year the prospects looked so dark.

On motion of Dr. Mollyneaux, a vote of thanks of the Association was extended to Dr. George Evans, of New York City, for coming to Cincinnati and delivering his interesting talk before the Association this morning.

Dr. J. Taft: I would suggest that a similar vote of thanks be passed in acknowledgment of the services of Dr. L. E. Custer, of Dayton, in connection with his demonstration of and improvements upon the electric oven.

The Chair stated the motion, after second.

Dr. Harlan, of Chicago: I think the expression toward Dr. Custer ought to be stronger than that. I think that Dr. Custer has done something for the dental profession which is deserving of a carefully-worded and well-written resolution, testifying our appreciation of his talents in this direction, and his generous intentions toward the Association, in presenting us freely and fully the fruits of his inventive genius in connection with the electric oven. I hope the Executive Committee, or some proper authority, will present it in that way. This is one of the bright stars in the galaxy of 1894, the discovery of the method of baking porcelain by electricity ; it is not inferior in any way to other great discoveries in electricity by men that are known the world over. (Applause.)

Dr. Mollyneaux: I move to amend to the effect that Dr. Taft write a suitable testimonial to Dr. Custer.

The amendment being accepted, the Chair put the question and the same carried by a unanimous vote.

The Chair appointed as a committee on publication, Messrs. Drs. H. T. Smith, J. Taft and E. G. Betty.

President Ames announced that he would appoint as Executive Committee, Drs. J. R. Callahan, M. H. Fletcher and L. E. Custer, to succeeed themselves. (Applause.)

Dr. Callahan: I am asking for an opportunity—

Dr. Cravens: I object. Nothing before the house.

Dr. Callahan: I would simply state that I would have positively refused any appointment of that kind, except from my friend from Chicago, whom I have a great desire to serve and will do the best I can. (Applause.)

Dr. J. Taft: In reference to the general meeting of all the societies of Ohio, Indiana and Michigan, to be held at Detroit in June, a cordial invitation is extended to all members to be present at that time; probably some of those present are not members of the State Society; but it is earnestly requested by the committee having the matter in charge that all persons connected with the societies of the three States, and other members of the profession as well, even though outside of the three States named, be invited to be present on that occasion, as guests. I may say that the profession in Michigan has decided to have all attending from outside of Michigan there as their guests. They have provided a liberal entertainment, and expect to have a grand occasion at that time; and the Ohio and Indiana, and all attending invited guests outside of those States, are to be the guests of Michigan; they are to provide all entertainment and pay all bills, so far as the entertainment is concerned. (Applause.) I feel warranted in extending a hearty invitation to all the members of this body to bring your wives, sweethearts, sisters and cousins, who wish to be present on that occasion; they want to make it an especially enjoyable occasion, and a good program is provided and the useful features will be very marked. They have provided a steamer which will go up the Detroit river to St. Clair flats, and they will have an excursion that will occupy a whole afternoon and evening; so there is no doubt but you will all have a good time. The social amenities will be a feature of the occasion. Michigan is anxious to have a large representation of the profession from this and neighboring States. (Applause.)

The list of delegates to the American Dental Association, as previously reported by the committee, was read and the action of the committee on motion confirmed, with the understanding that if others wished to go in that capacity they would be accepted up to the limit of the quota allowable from this Association.

On motion of Dr. J. Taft, the matter of time for the next meeting of the Association was referred to the Executive Committee with power to act, including the officers of the Association.

Dr. J. Taft: It has now been demonstrated that the time for the dissolution of this society has not come; but that it is fit to live a while longer. In view of the fact that we have had so successful a meeting I trust that every member will consider himself and herself a committee of one to see that the next annual meeting is not inferior to this. I must say that I have never seen a more interesting meeting or one into which more profitable work was crowded in two days than that of yesterday and to-day, never in any meeting that I have attended. We have no politics in this meeting and very little of unnecessary routine; just a few little matters in order to keep the machinery oiled for smooth running, in the way of miscellaneous business.

I trust that next year we will come with a determination to make the session of the Mississippi Valley Association equal to this in interest and as much better as possible. Let us all bear this in mind and do what we can to make the next annual meeting an eminent success.

Dr. Mollyneaux: I want to make a motion that somebody be appointed to get Dr. Callahan mad! Last year he tried to kill the Society because they sat down on him so hard, and this year it went the other direction. If somebody can get his dander up we will have a good meeting next year.

Dr. Callahan: If I remember rightly, Dr. Callahan did no such thing as that.

Dr. Wright: With all due modesty, I claim all the credit for this success this time.

On motion, adjourned to meet in Cincinnati, at a future date to be announced by the Executive Committee.

